# Targeting Intracellular Bacteria with Dual Drug-loaded Lactoferrin Nanoparticles

**DOI:** 10.1021/acsinfecdis.4c00045

**Published:** 2024-04-05

**Authors:** Moses Andima, Annette Boese, Pascal Paul, Marcus Koch, Brigitta Loretz, Claus-Micheal Lehr

**Affiliations:** † Department of Drug Delivery (DDEL), Helmholtz Institute for Pharmaceutical Research Saarland (HIPS), Helmholtz Centre for Infection Research, Campus E8.1, Saarbrücken 66123, Germany; ‡ Department of Chemistry, Faculty of Science and Education, Busitema University, P.O Box 236, Tororo 21435, Uganda; § Department of Pharmacy, Saarland University, Saarbrücken 66123, Germany; ∥ INM-Leibniz Institute for New Materials, Campus D2 2, Saarbrücken 66123, Germany

**Keywords:** intracellular bacteria, lactoferrin nanoparticles, targeted drug delivery, drug combinations, nanomedicine

## Abstract

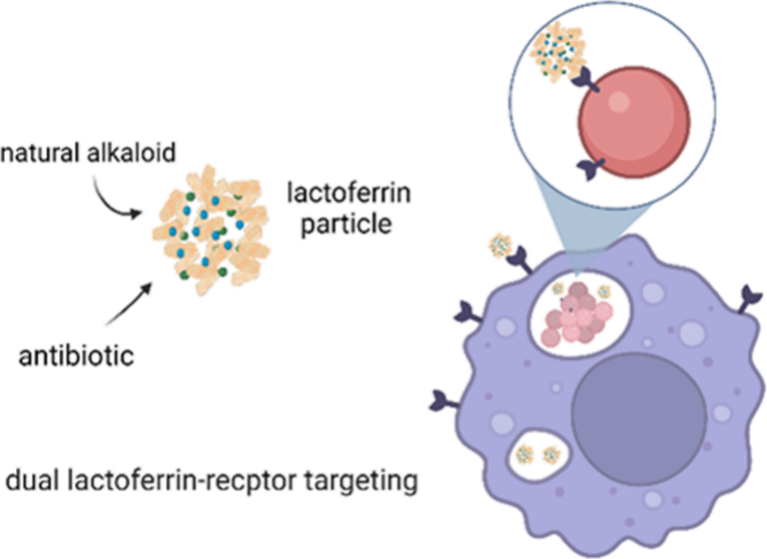

Treatment of microbial infections is becoming daunting because of widespread antimicrobial resistance. The treatment challenge is further exacerbated by the fact that certain infectious bacteria invade and localize within host cells, protecting the bacteria from antimicrobial treatments and the host’s immune response. To survive in the intracellular niche, such bacteria deploy surface receptors similar to host cell receptors to sequester iron, an essential nutrient for their virulence, from host iron-binding proteins, in particular lactoferrin and transferrin. In this context, we aimed to target lactoferrin receptors expressed by macrophages and bacteria; as such, we prepared and characterized lactoferrin nanoparticles (Lf-NPs) loaded with a dual drug combination of antimicrobial natural alkaloids, berberine or sanguinarine, with vancomycin or imipenem. We observed increased uptake of drug-loaded Lf-NPs by differentiated THP-1 cells with up to 90% proportion of fluorescent cells, which decreased to about 60% in the presence of free lactoferrin, demonstrating the targeting ability of Lf-NPs. The encapsulated antibiotic drug cocktail efficiently cleared intracellular *Staphylococcus aureus* (Newman strain) compared to the free drug combinations. However, the encapsulated drugs and the free drugs alike exhibited a bacteriostatic effect against the hard-to-treat *Mycobacterium abscessus* (smooth variant). In conclusion, the results of this study demonstrate the potential of lactoferrin nanoparticles for the targeted delivery of antibiotic drug cocktails for the treatment of intracellular bacteria.

Despite remarkable advances in human medicine, infectious diseases have remained a big public health threat, affecting millions of lives and causing substantial economic losses globally. Caused by bacteria, fungi, viruses, and parasites, the burden of infectious diseases ranks very high, particularly in developing countries.^1^ Antimicrobial drugs have only been partially successful in reducing microbial disease burden because microbial pathogens spontaneously develop resistance mechanisms to the available antimicrobial drugs.^2^ The need to search for novel therapies, on the one hand, and rethinking drug delivery strategies for existing therapies, on the other hand, is imminent. In the latter, appropriate drug delivery systems and smart formulation strategies need to be exploited to circumvent some of the resistance strategies adapted by pathogens.^3^

One of the strategies adapted by pathogenic bacteria to evade treatment is to localize in intracellular compartments making it difficult to eradicate them because many drugs fail to cross cellular barriers like the plasma and vehicle membranes in sufficient quantity.^4^ In the intracellular niche, the bacteria may also evade or even manipulate the host immune response enabling them to multiply and create a reservoir, which becomes a source of new infections.^4^ Targeted drug delivery systems that can circumvent intracellular barriers are required to deliver therapeutic payloads to eradicate intracellular bacteria.

Currently, the available antimicrobial drugs have failed to address the pitfalls associated with intracellular bacteria because of various limitations including, limited penetration into cells, poor retention inside cells, fast-pass metabolism, and degradation inside the target cells.^5^ Nanocarriers have been demonstrated to play a crucial role in overcoming the aforementioned challenges associated with intracellular bacteria.^6,7^ Foremost, the nanoscale size facilitates enhanced penetration into cells, allowing for efficient delivery of drugs to the intracellular bacteria.^6^ Moreover, nanocarriers can be tuned to selectively target infected cells and facilitate controlled release making them promising tools in the development of more effective and targeted antimicrobial therapies.^4,7^ Additionally, the nanoscale architecture can facilitate the codelivery of multiple therapeutic agents, thus enabling combination therapies that can target different phases of the bacterial life cycle to overcome drug resistance.^8^

Human cells and human pathogens alike require iron, an essential micronutrient for their survival.^9^ Besides high-affinity iron chelators (siderophores), it has been shown that some pathogenic bacteria express receptors for lactoferrin and transferrin (LbpB and TbpB) to capture iron from these host iron-carrying proteins.^9−12^ Interestingly, to increase their efficiency in capturing iron during infection, classical intracellular bacteria, such as mycobacteria, deploy receptors similar to the host surface receptors to sequester iron from host iron-carrying proteins. For instance, glyceraldehyde-3-phosphate dehydrogenase (GAPDH) has been demonstrated to act as a lactoferrin receptor for both human macrophages and some pathogenic bacteria.^13,14^ In this context, we reasoned that lactoferrin could be used for dual targeting of anti-infective drugs to macrophages and also to intracellular bacteria residing in macrophages.

For this study, we describe the development and evaluation of lactoferrin nanoparticles loaded with a dual drug combination, capable of targeting lactoferrin receptors expressed by macrophages and resident bacteria to eradicate the latter. Lactoferrin in addition to having both hydrophilic and hydrophobic regions on its surface can self-assemble into nanoparticles.^15^ This merit was exploited here to encapsulate both hydrophilic and hydrophobic drugs in a lactoferrin nanocarrier. We selected berberine (Berb) or sanguinarine (Sang) in combination with imipenem (Imip) or vancomycin (Vanco) (Supplementary Figure S1) for lactoferrin-mediated delivery to intracellular bacteria. Both berberine and sanguinarine are alkaloids that have shown potential as natural alternatives to traditional antibiotics in the treatment of infections caused by resistant microorganisms.^16−20^ They possess a large hydrophobic surface, which profits from hydrophobic interactions with the hydrophobic pockets of lactoferrin,^21,22^ thus facilitating efficient encapsulation and modulation of their release from the nanocarrier system. However, further research is needed to fully understand their mechanisms of action and to determine their effectiveness in combination therapy. We therefore investigated combinations of these two alkaloids with the two last-resort antibiotics, vancomycin and imipenem, to identify possible synergistic or additive effects against two pathogenic intracellular bacteria, *S. aureus* and *M. abscessus*. Vancomycin and imipenem are hydrophilic drugs that benefit from hydrophilic interactions such as hydrogen bonding with polar amino acids in lactoferrin to facilitate their inclusion in the nanoparticles. Lactoferrin nanoparticles as carriers for such drug combinations were prepared and applied to macrophage intracellular infection models to challenge the targeting concept.

## Results

### Combinations of Sanguinarine and Berberine with Imipenem or Vancomycin Exhibit Synergistic and Additive Effects against *S. aureus* and *M. abscessus*

Berberine and sanguinarine are plant-derived alkaloids, which exert broad antimicrobial activity by disrupting bacteria membranes, inhibiting DNA, RNA, and protein synthesis, and biofilm formation.^19,23^ First, we investigated whether combinations of berberine and sanguinarine with vancomycin or imipenem produce improved (synergistic or additive) activity against *S. aureus* and *M. abscessus*. We observed that a combination of sanguinarine with vancomycin was synergistic against *S. aureus* (Newman strain), producing a fractional inhibitory concentration index (FICI) of 0.29 and additive against the *M. abscessus* smooth variant (FICI > 0.5, Table 1). On the other hand, its combination with imipenem was synergistic against *M. abscessus* (FICI = 0.33) and additive against *S. aureus*. The combination of berberine with vancomycin was additive against *S. aureus* but its combination with imipenem was synergistic against *M. abscessus* (FICI 0.33). Overall, these results demonstrate the potential of berberine and sanguinarine for use in combination therapy against bacterial infections.

**Table 1:** Synergy Screen between Vancomycin, Sanguinarine, Berberine, and Imipenem Combinations against *S. aureus* and *M. abscessus* by Determination of Fractional Inhibitory Concentration (FIC) and the Fractional inhibitory Concentration Index (FICI)^a^

	*S. aureus*				*M. abscessus*			
**treatment**	**MIC (μg/mL)**	**MIC in combination (μg/mL)**	**FIC**	**FICI**	**MIC (μg/mL)**	**MIC in combination (μg/mL)**	**FIC**	**FICI**
vancomycin	1.25	0.156	0.125	0.29	>250	1.56	<0.10	>0.50
sanguinarine	12.50	2.0	0.160		125	62.50	0.50	
vancomycin berberine	1.25	1.25	1.00	0.29	>250	12.50	<0.10	>0.50
	250	3.91	0.02		>250	125.00	0.50	
imipenem sanguinarine	0.03	0.01	0.26	0.51	10.00	2.00	0.20	0.33
	12.50	3.12	0.25		125.00	15.63	0.13	
imipenem berberine	0.03	0.03	1.00	2.00	10.00	2.00	0.20	0.33
	250	250	1.00		>250	31.25	0.13	

a FICI ≤ 0.5 = synergy; 0.5 < FICI < 1.0 = additive; 1 < FICI ≤ 4.0 indifferent; > 4.0 = antagonism.

**Figure 1:**
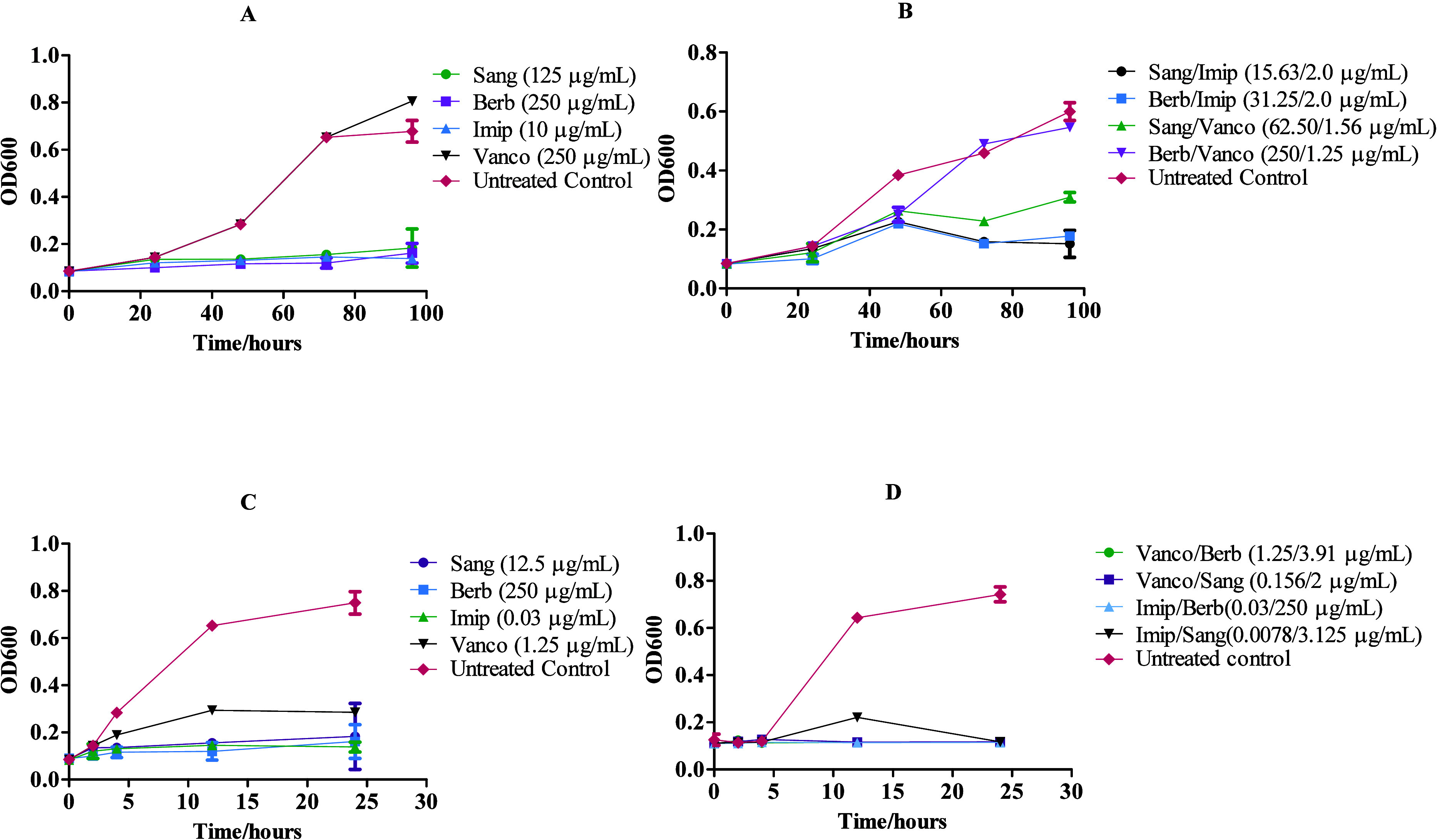
*In vitro* growth kinetics of *M. abscessus* (A, B) and *S. aureus* (C, D) when treated with single drugs (A and C) and drug combinations (B and D) at the determined MICs (*n* = 3 ± SD).

We further measured the growth kinetics of both investigated bacteria when treated with the drug combinations at concentrations equal to their MIC values. As a control, single drugs were included at their MIC. The results (Figure 1) demonstrate that all of the drugs individually inhibited bacteria growth at their MIC values except vancomycin, which did not show any inhibitory activity against *M. abscessus* at the highest concentration tested. Combinations of sanguinarine and berberine with vancomycin or imipenem inhibited bacteria growth over the incubation period. Surprisingly, when treated at their MIC, a combination of vancomycin and berberine was only bacteriostatic against *M. abscessus*. Taken together, berberine and sanguinarine could be used in combination with either vancomycin or imipenem against *S. aureus* and *M. abscessus* infections.

### Preparation and Physicochemical Characteristics of Lf-NPs

**Figure 2:**
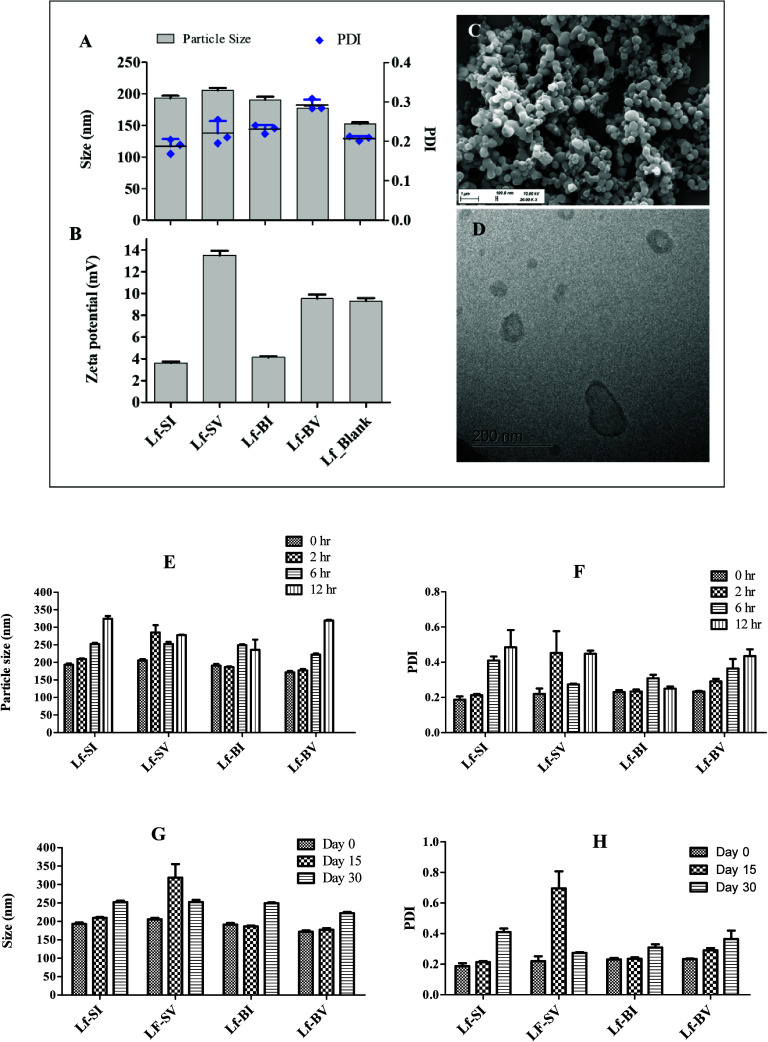
Characteristic properties of Lf-NPs as measured by DLS, SEM, and Cryo-TEM (A); particle size and size distribution (B); zeta potential (C, D); representative surface morphology as measured by SEM and cryo-TEM, respectively, (E, F); colloidal stability of Lf-NPs in PBS containing 10% FCS, (G, H) storage stability over 30 days at 4 °C. Values represent mean ± SD (*n* = 3).

Drug-loaded lactoferrin nanoparticles (Lf-NPs; subsequently labeled as Lf-SI, Lf-SV, Lf-BI, and Lf-BV for lactoferrin nanoparticles loaded with a combination of sanguinarine (S), imipenem (I), berberine (B), and vancomycin (V)) were prepared by a modified nanoalbumin bound (nab) method, whereby we included mPEG2000 to stabilize the nanoparticles in solution. Lf-NPs, as characterized by scanning electron microscopy (SEM), cryo-transmission electron microscopy (Cryo-TEM), and dynamic light scattering (DLS) (Figure 2A–D), were spherical with an average size of 191.8 ± 11.1 nm, uniform size distribution (PDI 0.23 ± 0.04), and positive zeta-potential (average 8.03 ± 3.84). Fluorescein (FITC)-labeled Lf-NPs as well as bovine serum albumin (BSA) nanoparticles used as controls for cellular uptake measurements exhibited similar physicochemical properties as the drug-loaded Lf-NPs and plain nanoparticles. The prepared lactoferrin nanoparticles were generally stable on storage over 30 days at 4 °C (Figure 2G,H). When evaluated in a physiological medium (phosphate-buffered saline (PBS) pH 7.4 containing 10% fetal calf serum (FCS)), there was a gradual increase in particle size and polydispersity index over 12 h (Figure 2E,F).

### Drug Encapsulation Efficiency and Drug Loading Capacity

As depicted in Table 2, Lf-NPs showed a high encapsulation efficiency with an average of 82.3% and drug loading capacity of about 1% (w/w). The actual amount of drug loaded (DL) for the investigated drugs was generally equal except for the combination of berberine and vancomycin (Lf-BV) where a decrease by a fifth in the actual amount of vancomycin loaded was observed. The high drug encapsulation efficiency is attributed to strong hydrophobic interactions of the drugs with hydrophobic regions of lactoferrin protein.^24^

**Table 2:** Encapsulation Efficiency and Actual Drug Loading of Lf-NPs^a^

formulation	encapsulation efficiency (%)	drug loading capacity (%)	actual amount of drug loaded (μg/mL)
Lf-SV	Sang 83.1 ± 0.62	1.03 ± 0.008	207.7 ± 1.56
	Vanco 87.9 ± 2.56	1.09 ± 0.031	219.7 ± 6.40
Lf-SI	Sang 82.9 ± 0.37	1.02 ± 0.005	207.2 ± 0.93
	Imip 84.7 ± 1.79	1.05 ± 0.022	211.8 ± 4.48
Lf-BV	Berb 85.5 ± 5.90	1.06 ± 0.073	213.8 ± 14.76
	Vanco 68.3 ± 1.84	0.84 ± 0.023	170.7 ± 4.60
Lf-BI	Berb 82.6 ± 1.79	1.02 ± 0.022	206.5 ± 4.48
	Imip 83.3 ± 0.34	1.03 ± 0.005	208.2 ± 0.85

a Data represents mean ± SD (*n* = 3 for three batches).

### Drug Release Profile

**Figure 3:**
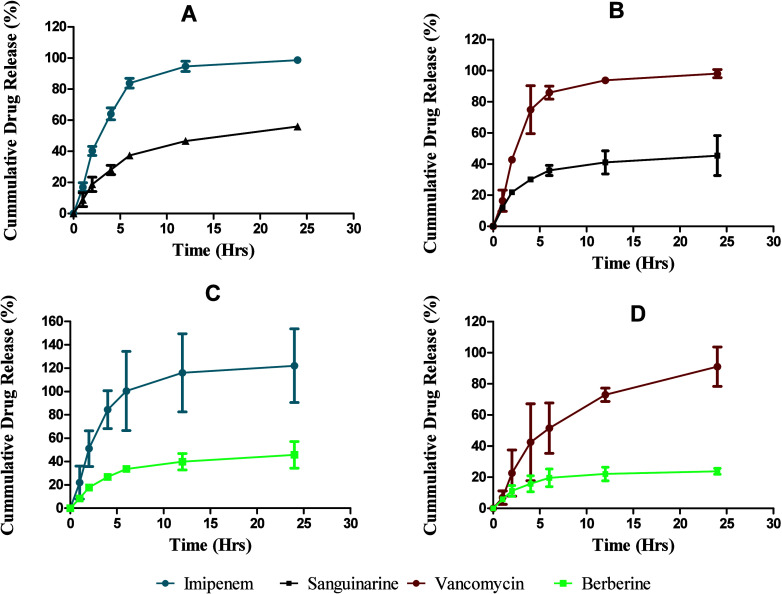
In vitro drug release profile of drug-loaded lactoferrin nanoparticles. (A) Lf-SI (B) Lf-SV, (C) Lf-BI, (D), and Lf-BV refer to lactoferrin nanoparticles loaded with combinations of sanguinarine (S), imipenem (I), berberine (B), and vancomycin (V), respectively, (*n* = 3 ± SD).

Lf-NPs exhibited gradual release of loaded drug at physiological pH (pH 7.4) over 24 h (Figure 3). However, there was a rather restricted release of sanguinarine and berberine from the nanoparticle matrix, which we attribute to strong hydrophobic interactions with hydrophobic regions of lactoferrin protein.^25^ In all cases, the drug release reached a plateau after 6 h followed by a slower release, where about 98% imipenem, 50% sanguinarine, 95% vancomycin, and 40% berberine were released within 24 h.

### Cytotoxicity Evaluation of Drug-Loaded Nanoparticles

**Figure 4:**
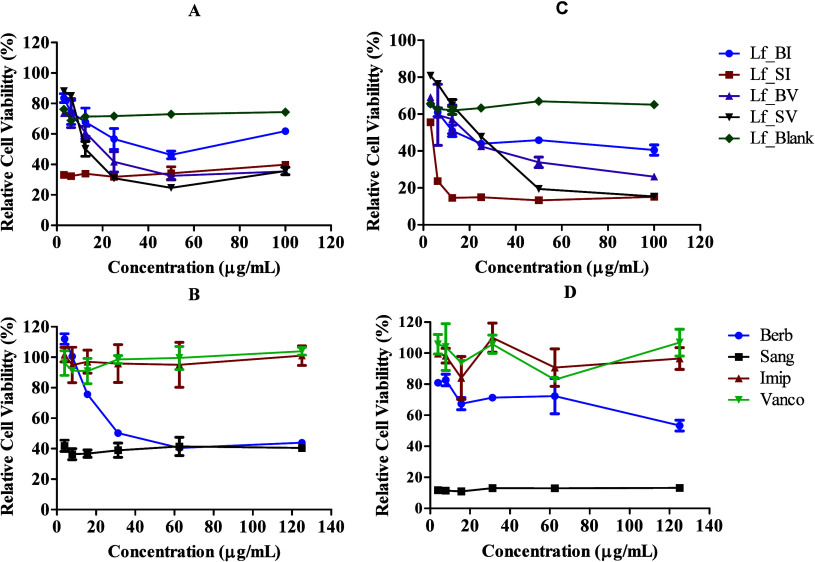
Graphical representation of viability of dTHP-1 (A, B) and A549 (C, D) cells when treated with varying concentrations of Lf-NPs and free drugs, respectively. Cells were seeded at a density of 1 × 10^5^ cells per well and treated with six serial dilutions of Lf-NPs (starting at ca. 100 μg/mL of the loaded drugs in nanoparticles) and the free drugs (starting at 125 μg/mL). Untreated cells were used as a negative control. Cell viability is expressed as a percentage of the control. Results represent mean ± SD (*n* = 3).

As a first indicator of safety, we investigated the cytotoxicity of free drugs and the drug-loaded Lf-NPs against A549 lung epithelial cells and differentiated THP-1 cells using the MTT assay. The results (Figure 4) show that all the drugs along with blank Lf-NPs were not very toxic to the treated cells except free sanguinarine and its combinations with either imipenem or vancomycin. Free sanguinarine inhibited cell viability by about 60% for epithelial cells and by over 80% for the more sensitive macrophages. However, it profits from its encapsulation in Lf-NPs where it exerts dose-dependent cell inhibitory activity with 70% viability at lower concentration levels. Toxicity profiles of sanguinarine and berberine have been reported in previous studies.^26^ Sanguinarine inhibits Na^+^ /K^+^ transmembrane protein, while berberine inhibits DNA synthesis in cells. Its inclusion in nanoparticles leads to a slow-release profile and this can be useful for targeted locoregional applications. Previous studies by Golla et al.^27^ showed that doxorubicin when encapsulated in lactoferrin nanoparticles did not have any significant toxicity in hepatocellular cancer animals despite the high toxicity of free doxorubicin.

### Lactoferrin Nanoparticles Show Enhanced Uptake by Differentiated THP-1 Cells

**Figure 5:**
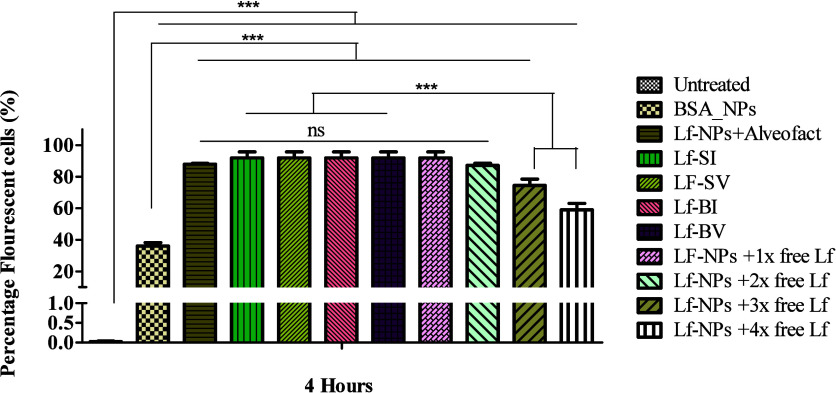
Quantitative FACS analysis of cellular uptake of Lf-NPs by dTHP-1 cells after 4 h of incubation. Cells were incubated with FITC labeled Lf-NPs at a concentration of 50 μg mL^–1^ in 48 well plates (total working volume of 300 μL) in the presence or absence of simulated alveolar surfactant or increasing concentration of free lactoferrin. Control groups included untreated cells and cells incubated with BSA nanoparticles at equivalent concentrations. Data represent mean ± SD (*n* = 3), significance is defined as *** (*P* < 0.001), ns = no significant variation.

Cellular internalization of Lf-NPs was studied by fluorescence-activated cell sorting (FACS) analysis after incubation of dTHP-1 cells with fluorescein labeled Lf-NPs for 4 h at 37 °C. Since macrophages express receptors for lactoferrin, we aimed to see the contribution of lactoferrin targeting for internalization of nanoparticles by macrophages. In particular, a pulmonary administration route would require efficient uptake even in the presence of lung surfactant and free lactoferrin present in lung lining fluid. Our results (Figure 5) show that about 90% of dTHP-1 cells efficiently internalized FITC-labeled Lf-NPs, which decreased to about 60% in the presence of increasing amount of free lactoferrin spiked in the culture media. The presence of pulmonary surfactant did not have a significant effect on the uptake of Lf-NPs (*p* > 0.05). To establish if the uptake of nanoparticles is independent of lactoferrin, BSA nanoparticles with similar physicochemical properties to Lf-NPs were incubated with dTHP-1 cells. Approximately 40% of dTHP-1 cells showed uptake of fluorescein-labeled BSA nanoparticles. Since BSA does not have specific receptors expressed by macrophages, in this case, its uptake by stimulated THP-1 cells is thought to be through nonspecific interactions. These results demonstrate specific targeting of lactoferrin nanoparticles to macrophages.

**Figure 6:**
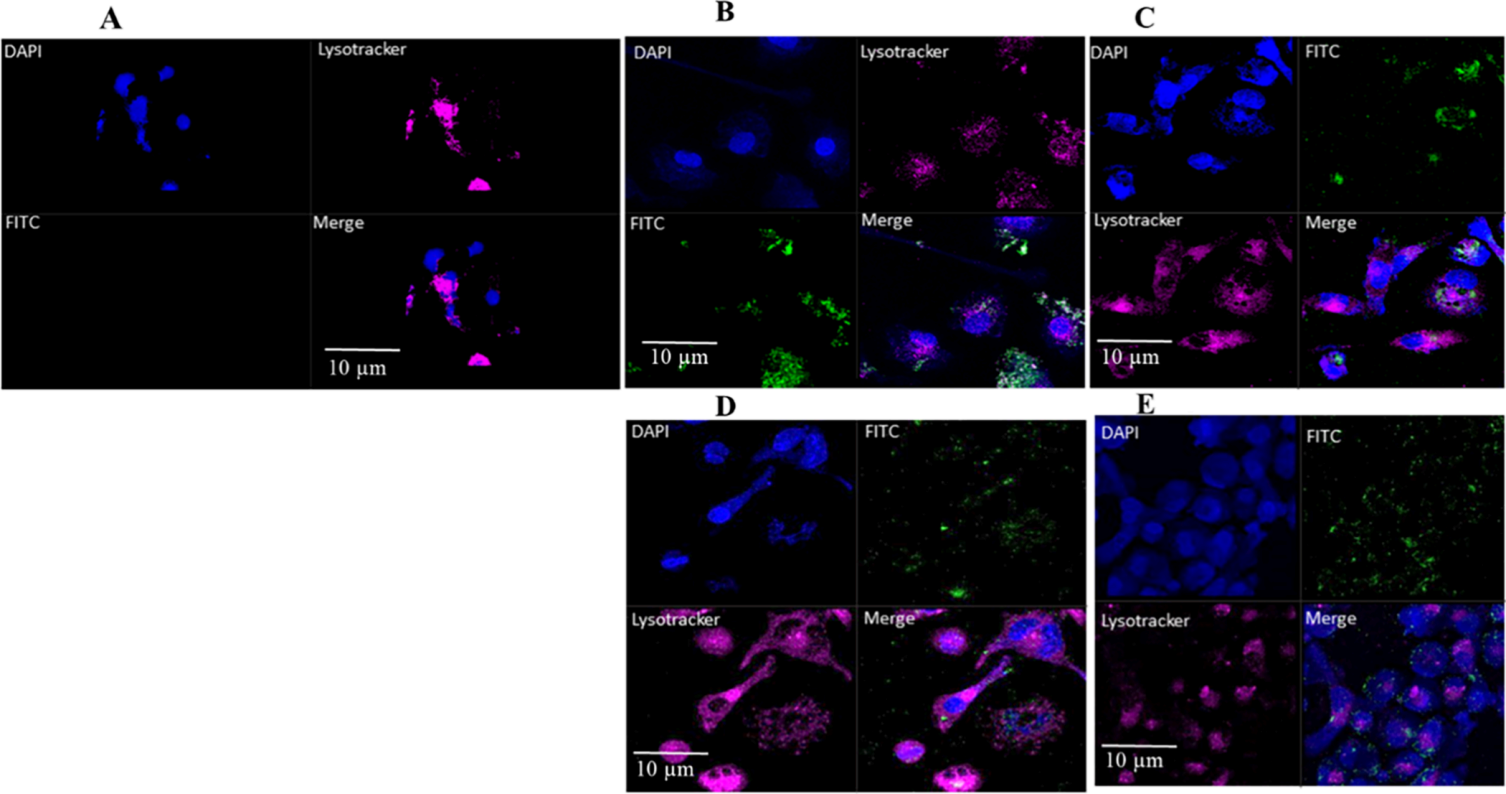
Intracellular distribution of FITC-labeled Lf-NPs; (B) Lf-SI, (C) Lf-SV, (D) Lf-BI, and (E) Lf-BV in dTHP-1 cells after 4 h incubation. Nuclei were stained with DAPI (blue) and lysosomes were stained with LysoTracker far-red. Controls (A) were labeled with DAPI and LysoTracker only.

To confirm flow cytometry measurements, we measured the fluorescence signals of the cells after treatment with fluorescently labeled Lf-NPs. The results (Figure 6) showed a green fluorescence signal of FITC-labeled particles internalized within the lysosomes labeled with LysoTracker far-red. This was not the case with the untreated control cells.

### In Vitro Antibacterial Activity against Extracellular Bacteria

Antibacterial activities of the free drug, drug combinations, and drug-loaded Lf-NPs were investigated by enumerating the number of colonies (CFU) post-treatment for extracellular and intracellular *S. aureus* and *M. abscessus*. In both cases, free drugs and drug combinations were applied at their MICs; meanwhile, Lf-NPs were applied at two final concentrations (50 and 25 μg/mL) to approximate the MICs of the drug combinations.

**Figure 7:**
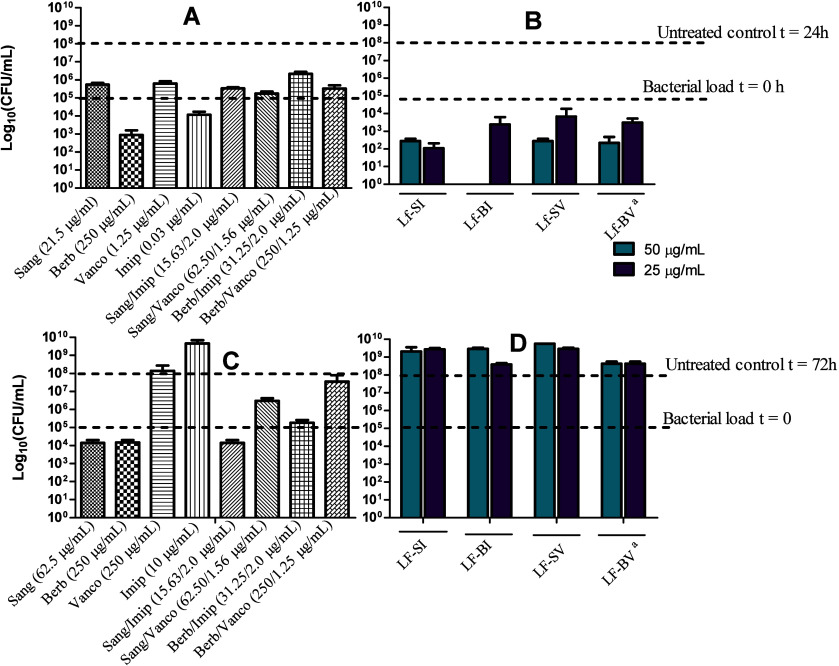
Antimicrobial activity of free drugs, drug combinations, and Lf-NPs against extracellular *S. aureus* (A, B) and *M. abscessus* (C, D). Bacteria were treated with the free drugs and drug combinations at their MICs. Lf-NP treatments were applied at two fixed concentration levels (final concentrations of 50 and 25 μg/mL) to approximate the MICs of the drug combinations. ^a^Concentrations of vancomycin were ca. 40 and 20 μg/mL).

For extracellular bacteria (Figure 7A,B), in comparison with the initial bacterial load, the free drugs and free drug combinations at their MIC generally exerted bacteriostatic effect against extracellular *S. aureus* except for berberine and imipenem, which showed 1–2 log reduction in the CFU counts. We observed that drug-loaded lactoferrin nanoparticles were more efficient in reducing colony counts for extracellular *S. aureus*, showing a 1–3 log reduction in CFU counts for most treatments and total clearance of extracellular bacteria by Lf-SI drug combination at a 50 μg/mL concentration level. Free drugs and combinations thereof generally produced bacteriostatic effects against extracellular *M. abscessus*. Surprisingly, imipenem and Lf-NPs were not effective in treating extracellular *M. abscessus*.

**Figure 8:**
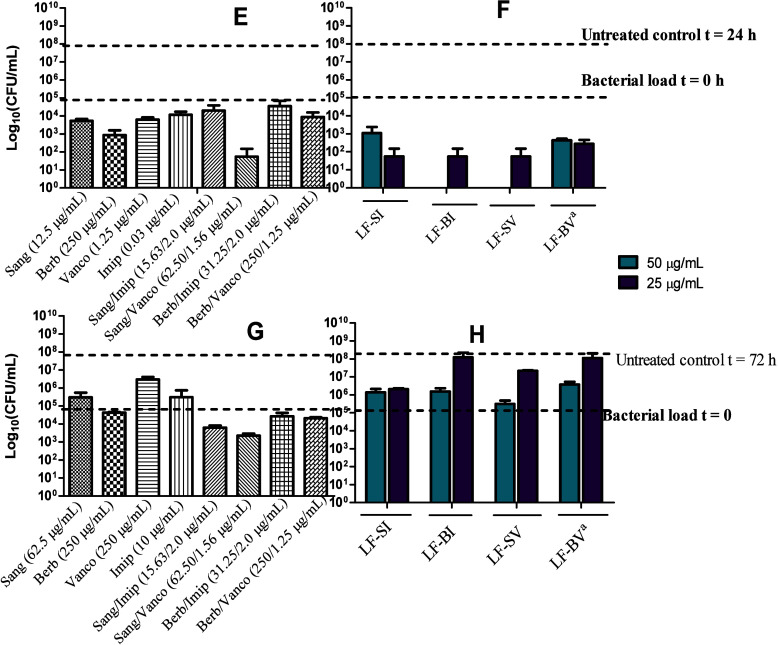
Antimicrobial activity of free drugs, drug combinations, and Lf-NPS against intracellular *S. aureus* (E, F) and *M. abscessus* (G, H). Bacteria were treated with the free drugs and drug combinations at their MICs. Lf-NP treatments were applied at two fixed concentration levels of 50 and 25 μg/mL to approximate the MICs of the drug combinations. ^a^Concentrations of vancomycin were ca. 40 and 20 μg/mL.

In the study of antibiotic effects on intracellular bacterial infections, it is crucial to inhibit the growth of extracellular bacteria to avoid false-positive or -negative results.^28^ In some cases, antibiotic protection is used to inhibit extracellular bacteria; however, a recent study^28^ shows that a sufficient quantity of the protecting antibiotic, mostly aminoglycosides, still penetrates and accumulates in the cells, primarily through pinocytic events, inhibiting intracellular bacteria. In this study, extracellular bacteria were eliminated from the intracellular infection model by consecutive washing steps using prewarmed PBS.^29^ The effectiveness of the washing steps was assessed in our preliminary experiments using light microscopy and confocal microscopy for FITC-labeled bacteria (Supporting Information S2), which showed efficient removal of extracellular bacteria. In addition, since some drug is released from the nanocarrier into the culture media, we reasoned that this would further inhibit any persistent extracellular bacteria. For intracellular bacteria (Figure 8E–H), Lf-NPs significantly inhibited intracellular *S. aureus* with total clearance of bacteria by Lf-BI and Lf-SV formulations at 50 μg/mL and up to 3 log reduction in colony counts for the other treatments. Free drugs and their combinations exerted generally bacteriostatic effects against intracellular *S. aureus*. All treatments were not very effective against intracellular *M. abscessus*.

## Discussion

Treatment of microbial infections is becoming a daunting task because many pathogenic bacteria evade treatment by developing resistance mechanisms, rendering the therapy ineffective. Unfortunately, the pace of development of new antibiotic agents is unmatched by the escalating need for antibiotics. Adoption of multidimensional strategies including structural modification of existing antibiotics, searching novel antibiotic drug scaffolds, exploring combination therapies, and development of robust antibiotic drug delivery systems able to circumvent some of the resistance mechanisms is much needed.^3,30,31^

In this study, we first carried out screening tests to evaluate potentially synergistic combinations of berberine and sanguinarine with imipenem or vancomycin against *S. aureus* and *M. abscessus*. It has been observed that drugs that act synergistically can overcome treatment failure even when bacteria resistant to one of the test drugs are present at the beginning of the therapy.^32^ Imipenem and vancomycin are last-line broad-spectrum antibiotics against multidrug resistant infections caused by Gram-negative and Gram-positive bacteria, respectively, especially in nosocomial infections. Unfortunately, resistance to these last-resort antibiotics has been reported in hospital settings,^33−35^ requiring strategies to overcome such resistance. Combination therapy is one of the strategies aimed at resensitizing resistant bacteria to these antibiotics.^33^ Sanguinarine and berberine are natural alkaloids that exhibit broad-spectrum antibiotic activity by destroying bacteria cell membranes, inhibiting synthesis of proteins and DNA, and biofilm formation^18,20,23,36−38^ and they have also been shown to potentiate other antibiotics.^20,39,40^ For instance, berberine in combination with linezolid, cefoxitin, and erythromycin showed significant synergistic activity against coagulase-negative Staphylococcus strains.^40^ In a separate study, Lu et al.^41^ demonstrated that sanguinarine synergistically potentiates aminoglycoside-mediated bacterial killing on diverse bacterial pathogens, including *Escherichia coli*, *Acinetobacter baumannii*, *Klebsiella pneumonia*, and *Pseudomonas aeruginosa* by increasing production of reactive oxygen species (ROS) and DNA damage.

Despite their vast applications for treating bacterial infections, the injection of vancomycin and imipenem results in negative side effects. For instance, the injection of high doses of vancomycin can lead to nephrotoxicity, ototoxicity, and hypersensitivity reaction; however, the injection of imipenem might cause gastrointestinal problems.^42,43^ In this study, we demonstrate that sanguinarine and berberine potentiate the activity of imipenem and vancomycin against *S. aureus* and *M. abscessus*, two important hard-to-treat bacteria responsible for nosocomial infections. When used in combination, berberine and sanguinarine lower the MIC of vancomycin and imipenem, which could be advantageous in reducing toxicity associated with using high doses. The combination of sanguinarine with vancomycin showed strong synergistic interaction (ΣFICI = 0.285) while other drug combinations were generally additive (0.5 < ΣFIC < 1.0).

Both *S. aureus* and *M. abscessus* are described as intracellular bacteria that can withstand the killing mechanisms of professional phagocytes, making it difficult to eradicate them.^44,45^ In nosocomial infections involving *S. aureus*, treatment options consist of the last-line drug vancomycin, which is administered invasively through the intravenous route. *M. abscessus* infections require a long-term phase treatment consisting of multiple drugs including clarithromycin-based multidrug therapy with amikacin and cefoxitin or imipenem often administered parenterally.^46^ In both cases, high doses of antibiotics are required to eradicate intracellular and multidrug-resistant bacteria,^4^ often resulting in off-target toxicity to noninfected organs and also to the associated useful microbiota^47,48^ and thus calling for some targeted drug delivery approach to eradicate intracellular bacteria more efficiently.

Lactoferrin-based nanocarrier formulations are promising options to deliver drugs to hard-to-treat intracellular bacteria and even more challenging cases such as biofilms.^49,50^ Their small size, ability to encapsulate both hydrophobic and hydrophilic drugs,^51^ and potential for targeted drug delivery^52^ are credited to this. Lactoferrin receptors are expressed on many cells including lung epithelial cells, macrophages, brain endothelial cells, liver cells, and cancer cells as well as some pathogenic bacteria, making them suitable to target therapeutic agents to these cells through receptor-mediated endocytosis.^52−54^*S. aureus* for instance has been shown to deploy receptors such as GAPDH, iron-regulated surface determinant protein (IsdA), and lactoferrin binding protein B (LbpB) among others^9−12,55−57^ to extract iron from lactoferrin and other host iron-carrying proteins. While there is scanty information about *M. abscessus* iron acquisition, mycobacteria in general have evolved mechanisms for iron acquisition, and lactoferrin is a potential source of iron for these bacteria.^13,58^ Recent studies have identified mycobactins as essential high-efficiency iron chelators deployed by *M. abscessus* to scavenge iron during intracellular growth within macrophages.^59,60^ Herein, a combination of the last-line antibiotics vancomycin and imipenem with naturally derived broad-spectrum antibiotic alkaloids, berberine and sanguinarine, were encapsulated into lactoferrin nanoparticles stabilized by mPEG200. We aimed at delivering the drug combination to eradicate intracellular bacteria by targeting lactoferrin receptors expressed by both macrophages and the bacteria (“one key for two doors” concept).

Lactoferrin is a cationic glycoprotein that can self-assemble into nanoparticles, or it can be conjugated onto the surface of other nanocarriers to target lactoferrin receptors. In our case, lactoferrin was self-assembled into nanoparticles with a PEG coating to ensure colloidal stability. PEGylation would be expected to significantly mask the surface of Lf-NPs consequently, reducing the targeting function, but this depends on the density of the PEG coating. Since Lf-NPs maintained an overall positive charge, the presence of a PEG coating would not significantly affect the targeting function. Our results demonstrate that dTHP-1 cells efficiently internalized Lf-NPs even in the presence of a simulated alveolar surfactant. Given the high basal phagocytic activity coupled with the targeting ability, pulmonary surfactant is expected to increase particle uptake by macrophages, while for epithelial cells, particle aggregation may rather reduce the uptake. Previously, pulmonary surfactant has been demonstrated to play a foe in nanoparticle uptake by alveolar epithelial cells,^61−63^ while other studies^64,65^ show the contrary. The difference between these studies is that the former used nanocarriers fabricated with materials that are not native to the pulmonary system, while the latter used native pulmonary surfactant to modify the nanocarriers. Since lactoferrin is present in lung fluids, the presence of pulmonary surfactant did not negatively affect the uptake of Lf-NPs. Interestingly, the proportion of Lf-NPs taken up by dTHP-1 cells decreased when free lactoferrin was included in the culture medium at increasing concentrations, which indicates specific uptake of lactoferrin nanoparticles. The current observation is similar to recent studies that utilized fucose as a targeting ligand on liposomal drug carriers.^29,66^ Confocal laser scanning microscopic analysis further confirmed the internalization of fluorescent Lf-NPs in the endosomal compartments. Although free lactoferrin (which is also present in the lung lining and other body fluids) reduced the uptake of Lf-NPs and given the high basal phagocytic activity of macrophages, it appears possible to administer decent quantities of Lf-NPs through the pulmonary route because high concentrations of Lf-NPs would be necessary for a competitive uptake of exogenous lactoferrin to achieve desired effects.

Following efficient uptake of Lf-NPs by dTHP-1 cells, we yearned to see whether this can translate to efficient eradication of intracellular bacteria. Indeed, Lf-NPs at final concentration levels of 25 and 50 μg mL^–1^ efficiently reduced the total colony count of *S. aureus* for both intracellular and extracellular bacteria. Lf-NPs loaded with combinations of berberine and imipenem (Lf-BI) and sanguinarine and vancomycin (Lf-SV) caused total eradication of intracellular *S. aureus* at a 50 μg mL^–1^ concentration level. Generally, the free drugs and free drug combinations caused only a 1–3 log reduction in the total colony count of *S. aureus* postinfection, thus demonstrating the benefit of targeted delivery achieved through lactoferrin nanocarriers. Earlier, Lehar et al.^67^ showed that an anti-MRSA antibodyantibiotic conjugate efficiently eradicated intracellular methicillin-resistant *S. aureus* (MRSA) infections compared to the free drugs. Similarly, when loaded into porous silica nanoparticles and conjugated to a cyclic 9-amino-acid peptide CARGGLKSC (CARG), vancomycin effectively suppressed intracellular *S. aureus* than free vancomycin.^68^ In the context of infectious diseases, these studies illustrate the potential of active targeting to eliminate intracellular infections.

Treatments against *M. abscessus* were not very effective in killing both intracellular and extracellular bacteria. In some cases, the treatments produced only bacteriostatic effects against *M. abscessus*. Infections due to *M. abscessus* are challenging to treat due to the widespread resistance of *M. abscessus* to many drugs, and its treatment often requires a combination of drugs administered over a long period.^69^ While MIC and growth inhibition assays showed that most of the treatments inhibited *M. abscessus*, in contrast, CFU determination revealed increased colony counts at the MICs especially for imipenem and vancomycin treatments. The discrepancy between OD600 measurements, indicating inhibition, and CFU determination, indicating bacterial viability, could arise from cell aggregation,^70^ delayed, and sublethal effects.^71^*M. abscessus* infections require long-term multidrug phase therapy. In the present study, cells infected with *M. abscessus* received a one-time treatment with Lf-NPs and free drugs and drug combinations, which only inhibited their growth without inhibiting viability, thus giving a higher colony count contrary to OD600 measurements. Future in vitro studies should focus on the phase treatment of infected cells for an effective clinical translation.

## Conclusions

Nanocarrier-based drug delivery coupled with active targeting provides a powerful tool to manage difficult-to-treat intracellular infections. In this study, we exploited the merit of pathogen-host relation in the war over iron mediated by host-iron-carrying protein, lactoferrin, to target intracellular bacteria with a dual combination of potentiating antibiotics loaded in lactoferrin nanoparticles. Lf-NPs exhibited desirable physicochemical parameters and favorable uptake by stimulated THP-1 cells, even in the presence of free lactoferrin, validating the targeting potential of the nanoparticles. The presence of obstructing pulmonary surfactant did not have a negative influence on the uptake of nanoparticles, showing potential for pulmonary administration. Drug-loaded Lf-NPs effectively eliminated intracellular *S. aureus* but not the hard-to-treat *M. abscessus*. Overall, this study demonstrates the potential of lactoferrin nanoparticles to deliver anti-infective agents to treat hard-to-treat intracellular bacteria. Additional studies will focus on optimizing Lf-NPs into microparticles for pulmonary administration of such anti-infective agents by oral inhalation.

## Materials and Methods

### Materials

#### Chemicals and Reagents

Human lactoferrin (iron saturated, ≥ 90%), sanguinarine (≥98%, HPLC), berberine (≥90% purity), vancomycin, imipenem (Pharmaceutical Secondary Standard), acetonitrile (>99.9% purity, HPLC), methanol (≥99.9% purity, HPLC), mPEG 2000, and all other chemicals were purchased from Sigma-Aldrich, Darmstadt, Germany.

#### Bacteria, Cells, Culture Media, and Supplements

*Mycobacterium abscessus* subsp. *Abscessus* smooth variant, isolated from the sputum of cystic fibrosis (CF) patients, was a kind gift from Prof. Dr. John Perry, Newcastle University and together with *Staphylococcus aureus* “Newman” MSSA: ATCC 25904), human leukemia monocytic cell line (THP-1, ACC 16, DSMZ, Braunschweig, Germany), and human lung adenocarcinoma basal epithelial cells (A549, ATC 107, DSMZ, Braunschweig, Germany) are maintained as freezer stocks in our laboratory. Middlebrook 7H11 agar, Middlebrook oleic acid-albumin-dextrose-catalase (OADC) supplement, Middlebrook 7H9 broth medium, brain heart infusion (BHI) agar, BHI broth medium, RPMI 1640 medium, phosphate-buffered saline (PBS, 10X) without calcium or magnesium, and fetal calf serum (FCS, Invitrogen) were purchased from Sigma-Aldrich, Darmstadt, Germany.

### Methods

#### Preparation of Lactoferrin Nanoparticles

Plain and drug-loaded lactoferrin nanoparticles were prepared by a modified nanoparticle albumin-bound technology.^72^ Briefly, human lactoferrin was dissolved in fresh milli-Q water to make a 40 mg/mL solution. Imipenem monohydrate and vancomycin were separately dissolved in Milli-Q water to make a 1 mg/mL drug solution and added to the lactoferrin solution constituting the aqueous phase. Berberine and sanguinarine were separately dissolved in an organic phase consisting of a solution of mPEG 2000 (2 mg/mL) in dichloromethane to give a final concentration of berberine and sanguinarine of 1 mg/mL. The organic solution was added to the aqueous solution and homogenized at 5000 rpm to form a crude emulsion. The crude emulsion was then transferred to a high-speed homogenizer (Kinematica polytron, Fisher Scientific) and homogenized at 18,000 rpm for 5 min. The organic solvent was evaporated by using a rotary evaporator under reduced pressure. The nanoparticle suspension was resuspended in milli-Q water and stored at −4 °C or lyophilized for further analysis. The particle formulations were subsequently labeled as Lf-SI, Lf-SV, Lf-BI, and Lf-BV representing lactoferrin nanoparticles (Lf) loaded with sanguinarine (S) or berberine (B) in combination with imipenem (I) or vancomycin (V). Bovine serum albumin (BSA) nanoparticles were prepared similarly for comparison.

#### Physicochemical Characterization of Lf Nanoparticles

Size, polydispersity index (PDI), and zeta potential of the optimized nanoparticle formulations were determined by dynamic light scattering (DLS) using a zetasizer ZS Series (Malvern Instruments Limited, Malvern, UK). The morphology of the particles was measured using a Zeiss EVO MA15 LaB6 field emission scanning electron microscope (Zeiss, Oberkochen, Germany) at 5.0 kV and 20,000× magnification and using a cryo-transmission electron microscope (Cryo-TEM) (JEOL, Akishima, Tokyo, Japan, model JEM-2100 LaB6).

#### Drug Encapsulation Efficiency and Loading Capacity

To determine the amount of drug encapsulated in Lf nanoparticles, the free drug was separated from the freshly prepared nanoparticle suspension (1 mL) using a centrifugal ultrafiltration unit (Centrisart, 10 kDa MWCO, Merck, Darmstadt, Germany) at 10,000*g* for 10 min. The amount of free drug in the supernatant was determined using an LC-MS consisting of an Ultimate 3000 ultrahigh-performance liquid chromatography (UHPLC) system coupled with a TSQ Quantum Access Max tandem quadrupole mass spectrometer (Thermo Fisher Scientific, Waltham, USA). Separation was achieved on an Accucore RP-MS C18 column (150 × 2.1 mm, 1.7 μm; Thermo Fisher Scientific) using a mobile phase consisting of acetonitrile (ACN, solvent A) and H_2_O solvent B both modified with 0.1% formic acid. Gradient elution was set up as follows: 10% solvent A for 2 min and a subsequent increase to 99% A for 8 min, which was then maintained for 3 min before a return to initial conditions. The mobile phase flow rate was 0.3 mL min^–1^ and the column oven was set at 40 °C. For mass spectrometric analysis, the fragment ion transitions were monitored using selected reaction monitoring (SRM) of the target compounds (sanguinarine [M]^+^ ion *m*/*z* = 332, with product ions 317, 304, and 274 *m*/*z*), berberine [M]^+^ ion *m*/*z* = 336.366 with product ions 321, 305, and 292, imipenem [M]^+^ ion *m*/*z* = 300 and product ions 282.075, 142.061, 124.112, and 96.185 *m*/*z*, and vancomycin [M]^+^ ion *m*/*z* = 724.981 with product ions 241.624, 100.071, and 72.190 *m*/*z*) using heated electrospray ionization (ESI) in the positive ion mode. The entire system was operated via the standard software Xcalibur (Thermo Fisher Scientific). The amount of drug loaded in the nanoparticles was determined as the difference between the amount of drug used in the nanoparticle formulation and the amount of free drug. Drug encapsulation efficiency (EE) and drug loading capacity (DLC) were calculated from the following equations
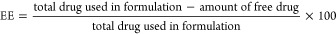
1
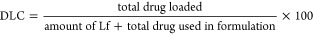
2

#### Drug Release Profile

In vitro drug release was investigated in HEPES buffer (pH 7.2) containing 50% methanol to maintain sink conditions for poorly soluble alkaloidal drugs. Briefly, 1 mL of nanoparticle suspension was placed in a slide-A-lyzer mini dialysis membrane (MWCO 10 kDa, Thermo Scientific, Waltham, MA USA) and inserted into 12 well plates containing 2 mL of the release medium. The setup was placed on a shaker maintained in an incubator at 37 °C. At predetermined time intervals, 400 μL of the release medium was withdrawn from the receiver compartment to determine the amount of drug released. At the same time, an equal volume of the fresh release medium was replenished in the receiver to maintain the volume of the release medium. The amount of drug released was assayed by using LCMS as described before.

#### Nanoparticle Colloidal Stability on Storage and in Physiological Medium

Stability of Lf-NPs was assessed on storage at 4 °C over 30 days. Colloidal stability was measured by dispersing the nanoparticles in a physiological medium consisting of PBS (pH 7.4) containing 10% FCS and incubating at 37 °C. Variations in particle size and surface charge were measured at specified time points.

### Biological Assays

#### Bacteria Strains and Culture Conditions

*S. aureus* working stock was prepared on brain heart infusion (BHI) agar medium at 37 °C for 24 h. Single colonies were then inoculated in 20 mL of BHI broth medium and incubated on a shaker at 37 °C for 18–24 h.

*M. abscessus* was inoculated on standard Middlebrook 7H11 agar medium containing 2.5 mL of glycerol supplemented with 10% OADC and incubated at 37 °C for 72 h to make a stock plate. Single colonies were inoculated in 20 mL of Middlebrook 7H9 broth medium containing glycerol and supplemented with 10% OADC and incubated on a shaker at 37 °C for 72 h, passaged, and incubated overnight.

#### Cells and Cell Culture Conditions

THP-1 cells, a human cell line displaying macrophage-like activity, were maintained in RPMI 1640 medium supplemented with 10% FCS, at an initial density of 2 × 10^6^ cells/ml at 37 °C in a humidified, 5% CO_2_ incubator. The cells were stimulated to differentiate into macrophages by adding phorbol 12-myristate 13-acetate (PMA, 10 ng/mL), in cell culture medium for 48 h. Differentiated THP-1 cells (consequently referred to as dTHP-1) were maintained in culture for a further 24 h period before use for bacterial infection and cytotoxicity assays.

Human lung adenocarcinoma basal epithelial A549 cells were cultured in RPMI 1640 medium supplemented with 10% FCS at an initial density of 2 × 10^5^ cells/mL in a humidified incubator maintained at 37 °C and 5% CO_2_^13^ and used for cytotoxicity studies.

#### Uptake Studies by Fluorescence-Activated Cell Sorting (FACS) Analysis

THP-1 cells were seeded at a density of 5 × 10^4^ cells/mL in 24 well plates, stimulated to differentiate into macrophages by adding phorbol 12-myristate 13-acetate (PMA, 10 ng/mL), and incubated for 48 h. Differentiated cells were washed with prewarmed PBS, treated with fluorescein-labeled Lf-NPs (final concentration of ca. 50 μg/mL based on the loaded drug), and incubated in RPMI 1640 medium for 4 h at 37 °C in a CO_2_ incubator with or without simulated alveolar surfactant (Alveofact, final concentration of 5 mg mL^–1^) or increasing concentration of free lactoferrin (final concentration 1–4× free lactoferrin). BSA nanoparticles and untreated cells served as controls. Thereafter, the cells were washed with prewarmed PBS and detached using 100 μL of trypsin solution for 10 min. Detached cells were washed twice with PBS and finally resuspended in PBS for FACS analysis using a BD LSRFortessa flow cytometer (BD Bioscience, San Jose, CA), and data was collected for 10,000 events and analyzed using FlowJo software, version 10.8.1 (FlowJo, Ashland, OR, USA).

#### Uptake by Confocal Laser Scanning Microscopy

To confirm flow cytometry measurements, cellular internalization was further measured by confocal laser scanning microscopy (CLSM). For this purpose, THP-1 cells were seeded at a density of 2 × 10^4^ cells per well in 8-well chamber slides and stimulated to differentiate into macrophages as described before. dTHP-1 cells were carefully washed with PBS and then incubated with FITC-labeled Lf-NPs at 37 °C for 4 h. The cells were further incubated with a fluorescent organelle-specific contrasting dye (Lyso Tracker deep red). After 1 h of incubation, the cells were washed thrice with sterile PBS and fixed with 4% paraformaldehyde (PFA) solution (200 μL) per chamber and incubated for 15 min. Cells were then washed with sterile PBS and incubated for another 20 min after staining with nuclei staining dye 4′,6-diamidino-2-phenylindole (DAPI) at a final concentration of 1 μg/mL (200 μL per chamber). The cells were finally washed with PBS twice and mounted using Fluorsave for confocal microscopy. Fluorescence images were acquired using a laser scanning confocal microscope (Leica SP8 inverted, Software: LAS X, Leica Microsystems GmbH, Wetzlar, Germany). The following were the image acquisition parameters: DAPI channel excitation at 405 nm, emission at 420–520 nm while FITC was excited at 488 nm, emission wavelength was set at 490–540 nm, and the Lysotracker deep red excitation wavelength was 633 nm, with emission at 640–700 nm.

#### Drug Susceptibility Tests

Broth microdilution assay was used to determine the antimicrobial susceptibility of the test organisms to the test compounds (berberine, sanguinarine, vancomycin, and imipenem). Briefly, 2-fold serial dilutions of test compounds (starting concentration 250 μg mL^–1^) were performed in clear, flat-bottom 96-well plates in 7H9-OADC medium for *M. abscessus* and BHI medium for *S. aureus*. Overnight cultures were diluted to an optical density measured at 600 nm (OD600) of 0.001 equiv to 10^5^ CFU/mL and added at equal volumes for a total volume of 100 μL per well in 96-well plates. The plates were incubated on a shaker at 37 °C for 72 h for *M. abscessus* and 24 h for *S. aureus* following the European Committee on Antimicrobial Susceptibility Testing (EUCAST) guidelines described in published literature.^73^ OD600 was measured after the incubation period against the untreated control. The minimum inhibitory concentration (MIC) was taken as the drug concentration at which there was no visible growth of bacteria (clear well) and was further confirmed by the Prestoblue viability reagent. The blue dye is reduced to a red/pink compound by active bacteria, where there is no growth of bacteria, and the reagent is not reduced (stays blue).

#### Checkerboard Assays

Potential synergistic interactions of the drug combinations between berberine or sanguinarine and the standard drugs imipenem and vancomycin were determined using standard 2D checkerboard assays. In this case, vancomycin or imipenem was serially diluted down the ordinate (B3–B10 in rows B-G) at a starting concentration 4 times higher than the final concentration in the wells. Berberine or sanguinarine, respectively, was diluted along the abscissa (column 3 row B to G) at a starting concentration 2 times higher than the final concentration in the wells. Column 2 from B2 to G2 and the first row from A3 to A10 contained individual drugs to provide the MIC for each drug alone. Columns 1 and 11 contained untreated controls, while column 12 and row H contained media blank. The plates were incubated as described before (at 37 °C on a shaker for 24 or 72 h, depending on the bacteria strain). OD600 was measured after the incubation period, then the PrestoBlue cell viability reagent was added, and the plates were incubated for a further 30 min for *S. aureus* and 3 h for *M. abscessus*. A visible color change from blue to pink/red indicated the growth of bacteria, and the visualized MIC was defined as the lowest concentration of drug combination that prevented growth (Prestoblue reagent remained blue). The sum of the fractional inhibitory concentration (ΣFIC) was used to define synergy. Here, the fractional inhibitory concentration (FIC) for each compound was calculated as
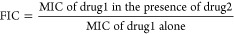
3

Then, the fractional inhibitory concentration index (FICI) was calculated as FIC1 + FIC2. The results were interpreted as follows: FICI ≤ 0.5 shows synergy, 0.5 < FICI < 1.0 shows additive effect, 1 < FICI ≤ 4.0 shows indifference, and FICI > 4.0: Antagonism.^74^

#### Cytotoxicity Assays

The MTT calorimetric assay was used to evaluate cytotoxicity profiles of the free drugs and drug-loaded-lactoferrin nanoparticles against dTHP-1 and A54 cells. Briefly, dTHP-1 and A549 cells were seeded at a density of 2 × 10^5^ cells/mL and incubated as described in the preceding section. The cells were treated with serial dilutions of the free drugs (concentration range of 125–3.91 μg/mL) and drug-loaded lactoferrin nanoparticle suspension (concentration range 100–3.125 μg/mL of active drug in nanoparticles) in cell culture medium. After 24 h of incubation, the media were aspirated and cells were washed once with sterile prewarmed PBS, treated with MTT dye (final concentration of 0.5 mg/mL per well), and then incubated for a further 4 h. Then, the culture medium was carefully removed, and the formazan crystals were dissolved using DMSO and incubated on a shaker for 30 min. Absorbance was measured at 570 nm using a plate reader (infinite Pro 200, Tecan, Switzerland).

#### Extracellular Antimicrobial Activity by CFU Determination

Free drugs and drug combinations were diluted in bacteria growth medium in 96-well plates to achieve concentrations equivalent to their MICs. Drug-loaded Lf-NPs were applied at two fixed concentrations (50 and 25 μg/mL of the loaded drugs) to approximate the MICs of the drug combinations. Overnight bacterial cultures, were diluted to an OD600 of 0.001–10^5^ CFU/ml and added at equal volumes for a total volume of 100 μL per well in the prepared 96 well plates. The plates were incubated as described before. After the incubation period, 10-fold serial dilutions of the contents of the wells were plated on 7H11 agar plates (for *M. abscessus*) and BHI agar (for S. aureus) in triplicate. Colonies were enumerated after 24 and 48 h for *S. aureus* and 72 days and 96 h for *M. abscessus*. Number of colony-forming units (CFU) per mL was calculated as
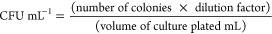
4

#### Intracellular Antimicrobial Activity by CFU Determination

For intracellular activity, THP-1 cells (2 × 10^5^ cells per well 400-μL final volume) were seeded in 24-well flat-bottomed tissue culture plates and differentiated into macrophages with 10 ng/mL PMA for 48 h and maintained in culture for a further 1 day. Bacteria growing at the log phase were harvested by centrifugation and washed twice with cold sterile PBS. The pellet was disrupted using glass beads on the vortex to generate single-cell bacilli. The bacteria concentration was estimated by measuring the OD at 600 nm, giving an OD600 of ∼0.5 equiv to 1 × 10^8^ CFU/mL. The bacterial cells were then suspended in RPMI medium with 10% FCS for 5 min for opsonization. dTHP-1 cells (2 × 10^5^ cells per well, 400 μL final volume) were infected with *M. abscessus* and *S. aureus* at a multiplicity of infection of 1:5 and 1:1, respectively. After 3 h, infected cells were washed carefully 3–4 times with sterile prewarmed PBS to remove extracellular bacteria.^29^ The cells were then treated with medium containing free drugs (at MIC), drug combinations (at MIC of combination), and Lf-NPs (final concentration of ca. 50 and 25 μg/mL of individual drugs) and incubated as before (24 h for *S. aureus* and 72 h for *M. abscessus*). Infected but untreated cells in the RPMI 1640 medium served as controls. Cells from three wells were lysed by adding Triton X-100 (0.05% in PBS), and the lysate was plated onto 7H10 or BHI agar medium to score CFU for untreated cells at time *T* = 0. After each time point, the cells were washed 3 times with PBS without Ca^2+^/Mg^2+^ and incubated in sterile PBS containing TritonX 100 (0.05% in PBS) for 30 min to lyse the cells and release the intracellular bacteria. Serial dilutions (1:10) of the lysate were performed and plated on 7H11 or BHI agar plates to enumerate the bacterial colonies.

### Statistical analysis

All experiments were done in replicate (*n* = 3) and the results represent mean ± standard deviation (SD) for replicate measurements. Statistical analysis and graphs were generated using GraphPad Prism version 5.0 (GraphPad Software, La Jolla, CA, USA 2007). Differences between groups were tested using one-way ANOVA followed by Tukey’s multiple comparison post-test analysis. Significance was defined as *** (*p* < 0.001) and ** (*p* < 0.005).

## Supporting Information Available

Figure S1: chemical structures of berberine, sanguinarine, imipenem, and vancomycin; Figure S2: micrographs of dTHP-1 cells infected with bacteria and after consecutive washing with prewarmed PBS to eliminate extracellular bacteria as visualized using light and confocal microscopes (PDF)
