# Supplementary material for: Targeting Intracellular Bacteria with Dual Drug-loaded Lactoferrin Nanoparticles
Source: ACS Infect Dis. 2024 Apr 5;10(5):1696–710. doi: 10.1021/acsinfecdis.4c00045 (PMC11091908; doi:10.1021/acsinfecdis.4c00045)
Supplement: Supplementary file 1 — id4c00045_si_001.pdf [file id4c00045_si_001.pdf]

SUPPORTING INFORMATION

Targeting Intracellular Bacteria with Dual Drug-loaded Lactoferrin Nanoparticles

Moses Andima [1,2], Annette Boese[1], Pascal Paul[1], Marcus Koch[4], Brigitta Loretz[1], Claus-Micheal Lehr[1, 3, *]

1 Department of Drug Delivery (DDEL), Helmholtz Institute for Pharmaceutical Research Saarland (HIPS), Helmholtz Centre for Infection Research, Campus E8.1, Saarbrücken 66123, Germany.

2 Department of Chemistry, Faculty of science and Education, Busitema University, P.O Box, 236, Tororo, Uganda.

3 Department of Pharmacy, Saarland University, 66123 Saarbrücken, Germany

4 INM-Leibniz Institute for New Materials, Campus D2 2, 66123 Saarbrücken, Germany.

* Address correspondence to: Claus-Michael Lehr**:** Department of Drug Delivery (DDEL), Helmholtz Institute for Pharmaceutical Research Saarland (HIPS), Campus E8.1, Saarbrücken 66123, Germany; https://orcid.org/0000-0002-5864-8462; Email: claus-michael.lehr@helmholtz-hips.de

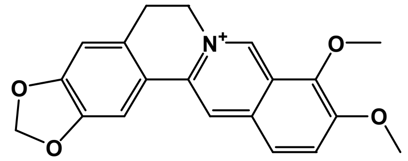

Berberine

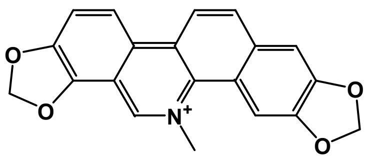

Sanguinarine

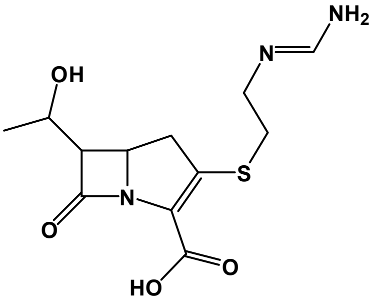

Imipenem

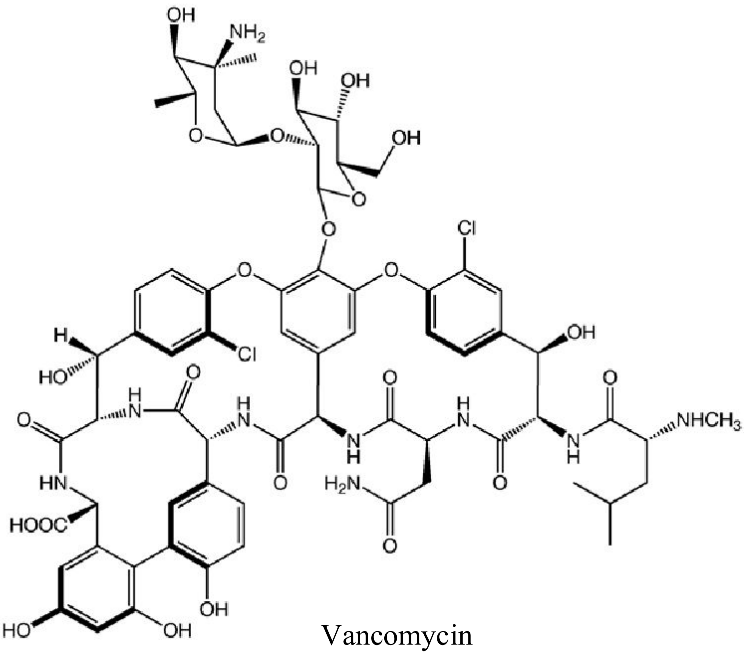

Vancomycin

Figure S1: Chemical structures of berberine, sanguinarine, imipenem and vancomycin

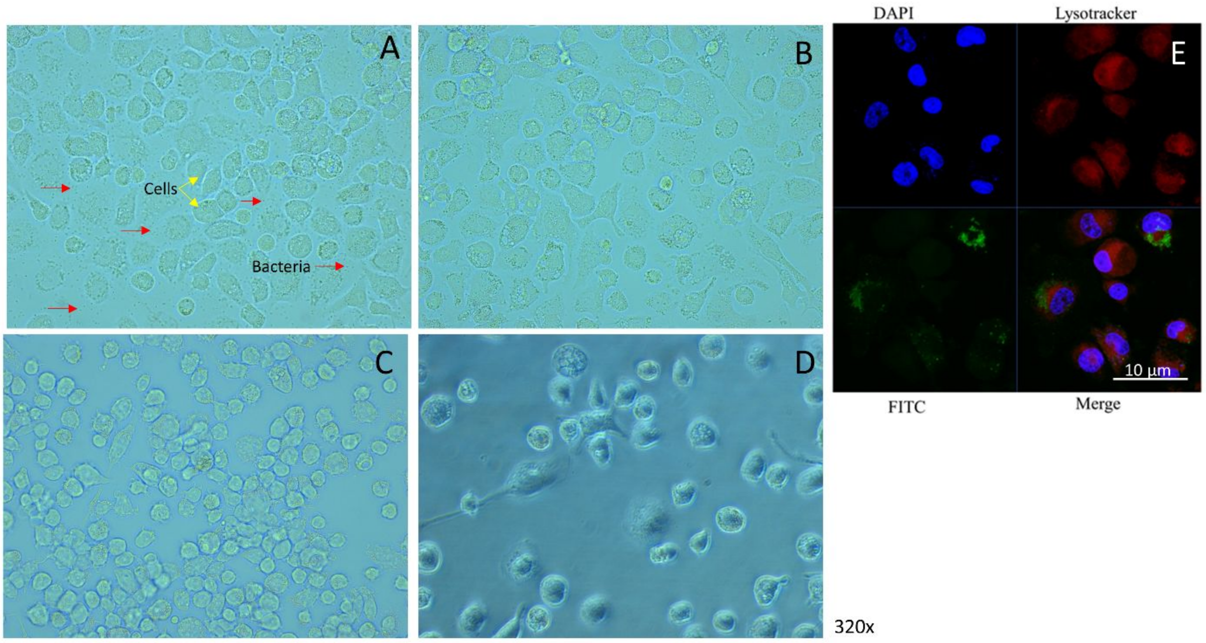

Figure 2: Representative micrographs of dTHP-1 cells infected with bacteria and after consecutive washing (3-4 times) with pre-warmed PBS to eliminate extracellular bacteria as visualized using light microscope (Carl Zeiss): (**A**) Infected cells before washing, (**B**) cells after 1x wash, (**C**) cells after 2x wash, (**D**) cells after 3x wash. Efficiency of washing steps was further evaluated after infecting cells using fluorescein labelled bacteria and visualized using confocal microscope (**E**). Cells were infected with 5/6-carboxyfluorescein succinimidyl ester (NHS-Fluorescein) labelled bacteria. The nuclei were stained with DAPI (blue) and lysosomes were stained with lysotracker red.
